# Insulin-Like Growth Factor 1, Glycation and Bone Fragility: Implications for Fracture Resistance of Bone

**DOI:** 10.1371/journal.pone.0117046

**Published:** 2015-01-28

**Authors:** Grażyna E. Sroga, Ping-Cheng Wu, Deepak Vashishth

**Affiliations:** Department of Biomedical Engineering and Center for Biotechnology and Interdisciplinary Studies, Rensselaer Polytechnic Institute, Troy, New York, United States of America; Queen’s University Belfast, UNITED KINGDOM

## Abstract

Despite our extensive knowledge of insulin-like growth factor 1 (IGF1) action on the growing skeleton, its role in skeletal homeostasis during aging and age-related development of certain diseases is still unclear. Advanced glycation end products (AGEs) derived from glucose are implicated in osteoporosis and a number of diabetic complications. We hypothesized that because in humans and rodents IGF1 stimulates uptake of glucose (a glycation substrate) from the bloodstream in a dose-dependent manner, the decline of IGF1 could be associated with the accumulation of glycation products and the decreasing resistance of bone to fracture. To test the aforementioned hypotheses, we used human tibial posterior cortex bone samples to perform biochemical (measurement of IGF1, fluorescent AGEs and pentosidine (PEN) contents) and mechanical tests (crack initiation and propagation using compact tension specimens). Our results for the first time show a significant, age-independent association between the levels of IGF1 and AGEs. Furthermore, AGEs (fAGEs, PEN) predict propensity of bone to fracture (initiation and propagation) independently of age in human cortical bone. Based on these results we propose a model of IGF1-based regulation of bone fracture. Because IGF1 level increases postnatally up to the juvenile developmental phase and decreases thereafter with aging, we propose that IGF1 may play a protective role in young skeleton and its age-related decline leads to bone fragility and an increased fracture risk. Our results may also have important implications for current understanding of osteoporosis- and diabetes-related bone fragility as well as in the development of new diagnostic tools to screen for fragile bones.

## Introduction

Insulin-like growth factor 1 (IGF1), a global growth regulator produced virtually in all tissues of a body, plays a central role in growth, development, and metabolism. The skeleton is a major depository organ of IGF1 [1, 2]. While tissue (paracrine) IGF1 is produced locally, serum (endocrine) IGF1 is produced in liver (nearly 75% of its total serum level). Both endocrine and bone-derived IGF1 contribute to normal longitudinal bone growth and cortical bone size as well as the maintenance of bone mass in adults [1, 3]. In humans, Langois et al. [4] observed a positive correlation between **serum** IGF1 and bone mineral density. Decreased serum levels of IGF1 were linked to an increased risk of osteoporotic fractures in humans [5]. Recent studies have demonstrated that low levels of IGF1 in **serum** were inversely associated with the fracture risk in older men, especially for the two important fracture types, hip and vertebral fractures [3, 6]. Currently there is no information available on the relationship between the levels of **bone-matrix** IGF1 and fracture toughness (i.e., bone’s ability to resist crack initiation and propagation) of human bone. We reasoned that the gradual decline of the IGF1 level in bone could not only slow down matrix turnover, but could also alter composition and quality of bone matrix, and ultimately, reduce bone fracture resistance.

The fracture of bone involves deformation and failure at multiple levels, which range from the nano- through micro- to macro-structural level [7]. To get insight into the role of IGF1 in bone’s resistance to fracture at the micro-structural level, we used the propagation-based fracture mechanics approach [8]. While initiation fracture toughness provides a measure of resistance against the initiation of a crack from a point of weakness in bone, the propagation fracture toughness provides a measure of fracture resistance against the propagation of pre-existing cracks, which initiate in bone during daily loading [9]. Therefore, measuring the propagation toughness not only allows more accurate characterization of bone toughness, but also directly relates to fracture [8].

Bone differs from all other tissues in a body by being composed predominantly of a mineral and a small amount of organic material that contains a uniquely large proportion of collagen. Based on this unusual composition, it is generally agreed that collagen plays a critical role in the structure and function of bone. In bone matrix, collagen fibers provide ductility (i.e., ability to deform under tensile stress) and ability to absorb energy (i.e., toughness). Alterations of collagen properties can therefore affect the mechanical properties of bone and increase fracture susceptibility. Several studies indicate that the large variation in bone strength may be in part related to differences in the quality of the collagenous matrix, including the nature and extent of its posttranslational modifications [10]. During synthesis and maturation, fibrillar collagens undergo numerous posttranslational modifications, which are performed by different enzymes. Enzymatic trivalent mature crosslinks such as pyridinoline (PYD) and deoxypyridinoline (DPD) are the predominant stabilizing inter- and intra-molecular crosslinks in mature tissues [10–13]. The formation of these crosslinks in the extracellular matrix is initiated by the conversion of telopeptidyl lysine and hydroxylysine residues to aldehyde form through the action of lysysl oxidase (LOX; EC 1.4.3.13, protein-lysine 6-oxidases) [14, 15]. The LOX-mediated crosslinking is crucial to the integrity and function of bone tissue. The content of PYD and DPD reaches a maximum concentration between 15–17 years of age in human bone and generally is maintained at a steady level over a lifetime [10–13] through the actions of IGF1 via enzymatic pathways [16–20]. In contrast to enzymatic crosslinks, non-enzymatic crosslinks vary with age. Consequently, our study is focused on non-enzymatic processes that lead to crosslinking of bone matrix proteins in healthy, normally aging donors.

In particular, it has been established that due to low turnover in a body, long-lived proteins accumulate advanced-glycation end-products (AGEs) and the accumulation levels of AGEs are high in diabetes and during aging [18, 21]. Formation of AGEs in bone accounts largely for the increase of collagen crosslinking after skeletal maturation. There are several molecularly well-defined AGEs that are specific to non-enzymatic glycation such as pentosidine [22], glucosepane [23] or vesperlisines [24]. Pentosidine (PEN) is a naturally fluorescent, mature crosslink of clinical relevance. It is commonly used as a biomarker of non-enzymatic glycation of bone matrix proteins. The measurements of PEN can predict vertebral strength *in vitro* independently of bone mineral density [25, 26]. It was also shown that PEN, quantified by high-pressure liquid chromatography (HPLC) [27], accounts for 9% of the variance in trabecular ductility [28]. In contrast, AGEs account for up to 40% of the cancellous bone fracture toughness [29]. Thus, in addition to quantifying PEN, we also measured AGEs “in-bulk” in order to determine the overall effects of AGEs and IGF1 on human bone cortical toughness. Studies involving healthy humans showed that IGF1, like insulin can function as one of several metabolic links between bone remodeling and energy metabolism. IGF1 increases glucose (a glycation substrate) uptake from a bloodstream in a dose-dependent manner [30, 31]. The pioneering work of Guler et al. [31] demonstrated that IGF1 enhances glucose metabolism directly as well as through circulating levels of insulin. Since the decline of IGF1 already begins around middle age and then progresses with aging [30], we reasoned that the positive effects of IGF1 on glucose metabolism would begin to diminish accordingly. We hypothesized that due to the association of IGF1 with glucose metabolism [31–33] the gradual decline of IGF1 would lead to lower bone matrix turnover and increase AGEs levels, and thus, would decrease bone’s resistance to fracture.

Currently there is no information on the relationship between fracture toughness of human bone and the extracellular bone-matrix levels of IGF1 as well as the levels of IGF1 in relation to fluorescent AGEs and pentosidine. Thus, the objectives of the present study were to examine whether the concentration of the matrix level of IGF1 in human cortical bone would associate with bone’s resistance to propagation toughness (crack growth resistance) and to explore if the decrease of IGF1 concentration would show a relationship with the content of fluorescent AGEs and/or pentosidine.

## Materials and Methods

### Human bone samples

Tibias (posterior area) from total of 33 human female donors (young 35.0 ± 15.0, middle age 60.0 ± 10 and elderly donors 80.0 ± 15.0 years old) served as the source of 41 cortical bone tissue samples. The specimens obtained from the centralized National Disease Research Interchange (NDRI) biobank were known to be free of osteoarthritis, diabetes and other metabolic bone diseases. They were also certified to be free of HIV and hepatitis B. All 41 samples were used for different biochemical analyses including samples selected for mechanical tests. Collected bone pieces were repeatedly washed in cold distilled water until the washings were free of blood, and then, defatted using isopropyl ether. After freeze-drying, the specimens were stored at -80°C until their use.

### Specimen preparation and mechanical testing

**Fig 1:**
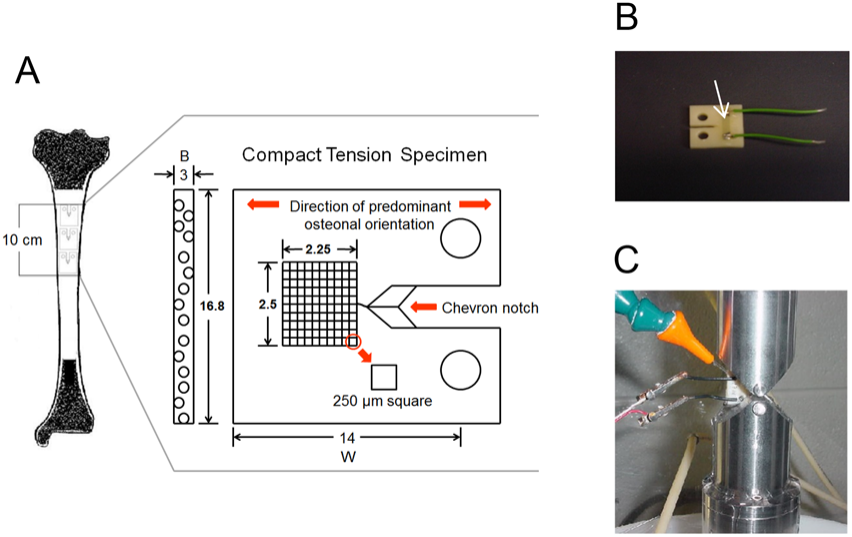
Compact tension specimen and mechanical testing. **A**. Schematic of a compact tension specimen and the area of human tibia used for preparation of the specimens. **B**. A picture of the compact tension specimen prepared for testing. The arrow shows the gauge, i.e., the crack propagation sensor that is present between the ends of the two wires. **C**. A cross-head of the MTS machine with a wetting outlet where the compact tension specimen was mounted for testing.

For mechanical tests, tibias of 20 cadaveric donors were selected (young 40 ± 6.0; middle age 60.0 ± 10.0 and elderly donors 80.0 ± 15.0 years old) and compact tension specimens from the mid-diaphysis of each of the donor’s tibia were prepared as shown in Fig. 1.

Each bone was cut into shapes of nominal dimensions 16.8 mm x 14.0 mm x 3 mm using a low speed diamond saw (Isomet 11–1180; Buehler, Lake Bluff, IL) and machined into miniaturized compact tension specimens (Fig. 1*A*). The chevron notch and holes were made using Denford CNC Micromilling machine. After machining, a crack propagation gauge (TK-09-CPB02, Micro-Measurements, Northboro, MA) was mounted on each specimen in order to monitor the increase of the crack length during testing (Fig. 1*B*). Subsequently, a given specimen was mounted onto a MTS 858 Mini Bionix II System (MTS Systems Corporation, Eden Prairie, MN) (Fig. 1*C*) and tested under wet conditions with a cross-head speed of 0.05 mm/min. Loading of the specimen resulted in the initiation of a crack from the notch. The crack was propagated parallel to the long axis of osteons. Changes in the gauge resistance corresponded to the measured change in voltage and specific crack length. For all tests, the fracture was allowed to propagate to the end of the gauge. After the load (*P*) and crack length (*a*) were acquired, the stress intensity factors at initiation (*K*_*IC*_, t = 0) and at each subsequent crack length (*K*_*R*_, t = 1, 2, 3…) were calculated according to the methods well described in literature [8, 34, 35].

### Determination of the IGF1 concentration by enzyme-linked immunosorbent assay

To determine the concentration of IGF1, the bone matrix from a total of 38 samples was isolated. Our goal was to analyze as many samples as we could get in order to address an important issue of natural variation in the levels of different biomolecules in a human body. The 38 donors represented 27 different ages. Thus, two samples originated from the same donor of age 19, 25, 29, 34, 48, 62, 76 and 97. In addition, one sample from a different donor of age 19, 34 and 76 was also analyzed. Bone pieces (0.030–0.050 g in size) were washed several times in cold distilled water, defatted using isopropyl ether, immersed in liquid nitrogen and ground into small particles (40–60 μm). A portion of each powder was taken out to determine DNA concentration as DNA is of cellular not matrix origin. A lack of DNA detection (i.e., less than 25 ng/0.010 g of bone powder) is treated as no contamination from cellular and blood components in the bone powder. Another portion of each powder was taken for direct hydrolysis in 6 N HCl. The remaining bone powders were transferred to eppendorf tubes that had a hole melted in the lid and then extraction buffer (0.05 M EDTA, 4M guanidine chloride, 30 mM Tris-HCl, 1 mg/ml BSA, 10 ul/ml Halt Protease Inhibitor, pH 7.4) was added. The tube was covered with a dialysis membrane (Spectra Por 3 Dialysis Membrane; Spectrum Laboratories, Inc., CA, USA) and closed allowing the membrane to line the hole. The micro-dialysis was conducted at 0–2°C against several changes of the PBS buffer pH 7.4. After dialysis, the samples were acidified to a pH of 2.3 (on ice in a cold-room). Under these conditions, IGF1 complexes dissociate, and IGF1 becomes accessible to antibodies [36]. Notably, collagen solubilizes at low pH (range from 2.1 to 6.8; here, pH 2.3) and releases any bound proteins and peptides into the solution. After releasing IGF1 from the complexes, the samples were centrifuged and a 25-fold excess of insulin-like growth factor 2 (IGF2) (Invitrogen/Life Technologies, Grand Island, NY) was added to the supernatants to block the re-binding of IGF1 to IGF Binding Proteins (IGFBPs) during the subsequent neutralization of each reaction mixture.

Detection and quantitation of IGF1 was performed using multispecies IGF1 ELISA kit (Ibt-GmbH, Reutlingen, Germany) according to the protocol included with the kit.

### Measurement of fluorescent AGEs

The samples were collected from a total of 31 donors representing 21 different ages. Thus, two samples originated from the same donor of age 19, 25, 29, 34, 48, 62, 76 and 97. In addition, one sample from a different donor of age 19 and 76 was also analyzed.

Direct acid hydrolysis of bone pieces (5–10 mg of a bone) was performed in 6N HCl (100 μl/mg bone) at 110°C for 20 hrs. After cooling, the hydrolysates were centrifuged, the respective supernatants were divided into portions, transferred to clean tubes, lyophilized and used for assays or stored at -80°C until use.

The fluorescent AGEs (fAGEs) assay has two components. The first component is a fluorometric assay for determination of fAGEs content. This assay is based on the measurement of natural fluorescence of AGEs as well as the fluorescence of the quinine standards (stock: 10 mg/mL quinine per 0.1 N sulfuric acid) at 360/460 nm excitation/emission using a microtiter-plate (MT-plate) reader (model Infinite 200; Tecan). The second assay component is a colorimetric assay for determination of collagen content in bone samples through the measurement of hydroxyproline (ProOH) concentration.

For ProOH assay, all solutions are made fresh. The assay was started by addition of chloramine-T to hydroxyproline standards (stock: 2 mg/mL *L*-hydroxyproline per 0.001 N HCl) and to the hydrosylates of bone samples. The resulting solutions were incubated at room temperature (RT) for 20 minutes. Subsequently, the 3.15 M perchloric acid solution was added to the reaction mixtures and the samples were incubated for 5 minutes at RT. Next, the *p*-dimethylaminobenzaldehyde solution was added and the samples were incubated for 20 minutes at 60°C. Finally, all standards and samples were cooled down to RT in darkness for 5 minutes. The absorbance was measured at 570 nm using the MT-plate reader. Collagen content was calculated based on the determined amount of ProOH as mmol collagen per mass of undemineralized bone [37]. Fluorescent AGEs were expressed in terms of unit of fluorescent quinine per unit of collagen.

### Measurement of pentosidine, pyridinoline and deoxypiridinoline by ultra-high performance liquid chromatography

Determination of the amount of PEN, PYD and DPD was conducted using ultra-high performance liquid chromatography (UPLC) [37, 38]. Two analyses were performed on each bone hydrolysate, one to measure pentosidine content, and a second to determine hydroxyproline content that was further used to calculate collagen concentration.

Before the UPLC analysis, each hydrolysate was dissolved in 1% *n*-heptafluorobutyric acid (HFBA). PEN was separated using an Acquity UPLC machine (Waters Corp., Milford, MA, USA) equipped with the reverse-phase Acquity UPLC HSS T3 column (1.8 μm; 2.1 x 100 mm). The column flow rate and temperature were 0.400 ml/min and 40°C, respectively. Solvent A contained 0.06% HBFA in 18 ohms pure water, and solvent B was composed of 50: 50 (v: v) mixture of solvent A: acetonitrile. Prior the use, the column was equilibrated using 10% solvent B. Gradient of 10 to 50% of solvent B was used for the separation of PEN, PYD and DPD. The elution of PEN was monitored for fluorescence emission at 385 nm after excitation at 335 nm (S1*A* Fig.). The elution of PYD, DPD and INT-PYD (a standard) was monitored for fluorescence emission at 395 nm after excitation at 297 nm. PEN, PYD and DPD were quantified using the standard curves. For additional information on PYD and DPD please refer to the supporting information.

### Measurement of hydroxyproline by UPLC

Hydroxyproline content was determined using reagents from the HPLC assay kit (Bio-Rad Labratories GmbH, Müchen, Germany), but the mobile phase solvents and conditions were developed specifically for the UPLC separation. The column flow rate and temperature were 0.400 ml/min and 60°C, respectively. The 0 to 50% gradient of acetonitrile was achieved by mixing 100% acetonitrile (solvent B) with a buffer composed of 0.3% acetic acid and 0.6% triethylamine, pH 4.50 (solvent A). The elution of the derivatized hydroxyproline was monitored at 471 nm (S1*B* Fig.). The amount of hydroxyproline was determined using standard curve. The amount of collagen was calculated assuming 300 nmol of hydroxyproline in 1 mol of collagen.

### Statistical analysis

All relationships were determined using simple linear and multiple linear regression analyses. Simple linear regression analyses were performed using MS Excel Statistical Analysis ToolPack. Multiple regression analyses were performed using the SAS/STAT software, ver. 9.1 executed on XP_PRO platform (SAS Institute, Inc., Cary, NC, USA). Results are expressed as means (± SD). Relationships between continuous variables were determined using linear regression. Multiple regression was performed for K_IC_ and for K_R_. For each response variable the explanatory variables were IGF1, fAGEs, PEN and age. Fluorescent AGEs and PEN were evaluated using IGF1 and age as explanatory variables. In addition to the multiple regression analyses, adjustment for age was performed using an added variable plot (partial regression plot). Added variable plots were evaluated to identify influential data points. In all analyses, p values less than 0.05 were considered statistically significant.

## Results

### Age-related decrease of matrix-bound IGF1 level in bone

**Fig 2:**
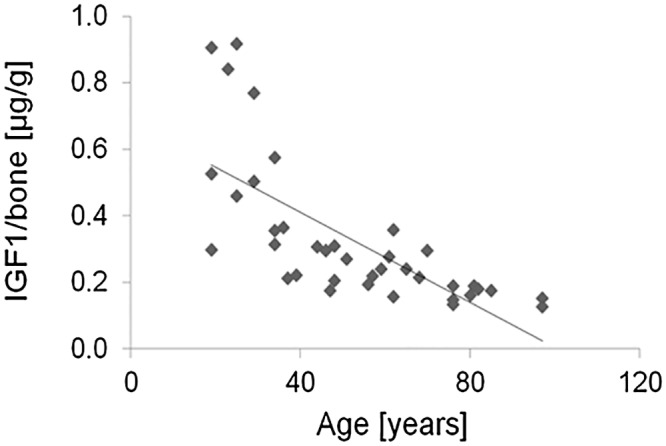
Concentration levels of IGF1 in bone matrix. The levels of bone-matrix IGF1 decrease with the increasing age of human donors (p<0.001).

We determined that the concentration of IGF1 in human tibial posterior cortex was inversely correlated with the age (Fig. 2; p<0.001; R^2^ = 0.51). The levels of matrix-bound IGF1 ranged from 0.114 ± 0.014 (elderly donors) to 0.902 ± 0.013 (young donors) μg/g bone (the ± values refer to the precision, i.e., to the measure of the repeated results variation of the IGF1 levels determined for three sample replicates) in the tested samples. Notably, the highest level of IGF1 was observed for one of the 19 year-old donors (the average calculated from the values of three independent measurements was 0.713 μg/g bone). Conversely, the lowest level of IGF1 was observed for 97 year-old donor (the average calculated from the values of three independent measurements was 0.223 μg/g bone). The determined levels of IGF1 per g of bone agreed with those published for other skeletal sites [39, 40]. Concentration of IGF1 in the tenth decade was on average about 3.2-fold lower than in the third decade of human life. Thus, we confirmed that similarly to other tissues, the levels of matrix-bound IGF1 in bone indeed decreased with the increasing age of the donors.

### IGF1 levels significantly associate with the accumulation of fluorescent AGEs

Two statistical analyses, a simple and a multiple linear regression analysis, were performed in order to determine the contribution of each parameter of interest (i.e., concentration of IGF1, AGEs and age) to the observed relationships.

**Fig 3:**
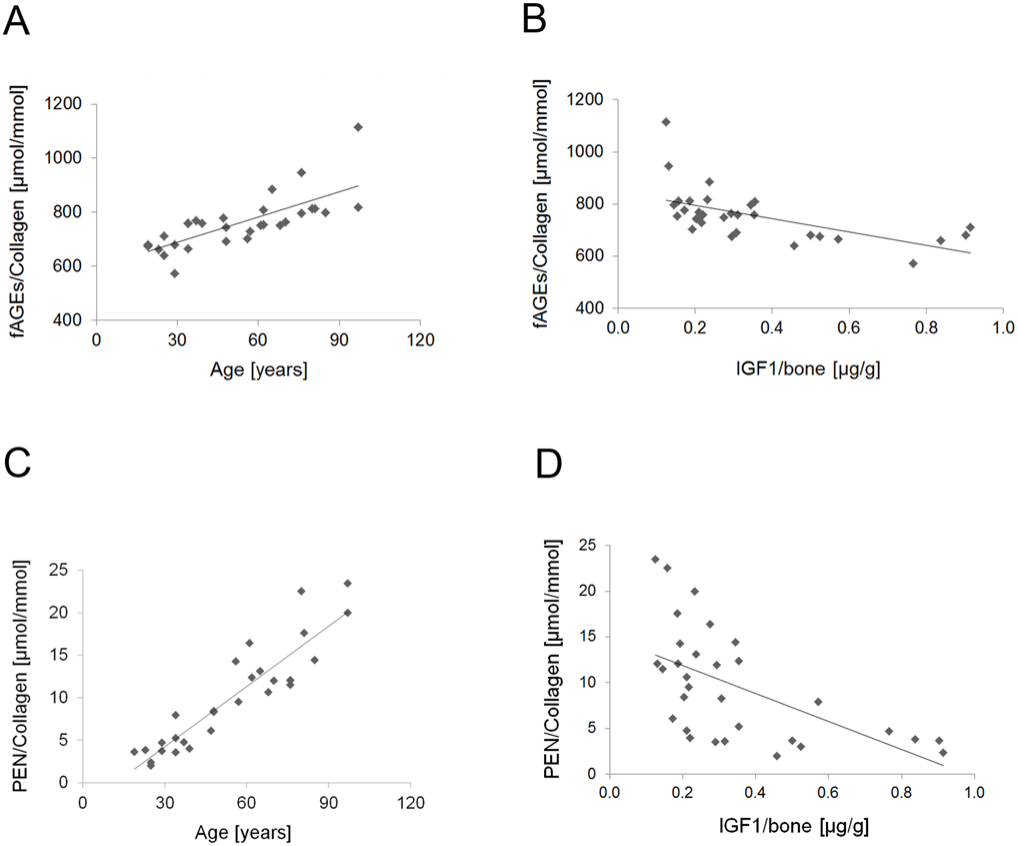
Associations observed between bone matrix IGF1, fluorescent AGEs and pentosidine. **A**. The levels of fAGEs (expressed as the concentration of fluorescent quinine per mmol collagen) increase with age of human donors (p<0.001). **B**. Increase of the fAGEs levels associates with the corresponding decrease of the bone-matrix IGF1 concentration (simple linear regression p<0.001; multiple linear regression p<0.04). **C**. Bone-matrix levels of PEN decrease with the age of human donors (p<0.001). **D**. The levels of PEN show inverse relationship with the concentration of bone-matrix IGF1 (simple linear regression p< 0.01 only).

First, we showed that the accumulation of fAGEs **normalized to the collagen contents in bone** increased with the donors’ age (Fig. 3*A*; p<0.001; R^2^ = 0.56). Generally, the amount of fAGEs was within the range of 572.5 ± 0.9 to 758.3 ± 0.3 μmol of fluorescent quinine units **per mmol of bone collagen** (calculated as average from the values of three independent measurements) in the donors until the age of approx. 40 years. In the older donors (50 years and older), the amount of fAGEs comprised from 778.0 ± 0.3 to 1113.9 ± 0.3 μmol of fluorescent quinine units per mmol of bone collagen.

The simple linear regression analysis revealed an inverse relationship between the levels of fAGEs/mmol of collagen and the IGF1 concentration (Fig. 3*B*; p<0.001; R^2^ = 0.35). Since concentrations of fAGEs and IGF1 are age-dependent, to confirm the relationship of fAGEs and IGF1, the multiple linear regression analysis was performed. In this analysis the response variable was fAGEs and the predictor variables were IGF1 and age. The analysis demonstrated that the accumulation of fAGEs was clearly associated with the decrease of IGF1 independently from age (p<0.04).

### Relationship between IGF1 and accumulation of pentosidine is influenced by age

The accumulation of PEN increased with the donors’ age (Fig. 3*C*; p<0.001; R^2^ = 0.83). The amount of PEN was within the range of 1.99 ± 0.04 to 8.40 ± 0.03 μmol/mmol of bone collagen in the donors until the age of approx. 40 years. Bone matrix from the donors 50 years and older contained 9.51 ± 0.03 to 23.45 ± 0.02 μmol PEN per mmol of bone collagen.

The simple linear regression analysis revealed that the accumulation of PEN was inversely related with the IGF1 concentration (Fig. 3*D*; p<0.01; R^2^ = 0.33).

To adjust the observed relationship between IGF1 and PEN for age, a multiple linear regression analysis was performed. This analysis revealed that unlike the association between fAGEs and IGF1, the relationship between the levels of PEN and IGF1 was influenced by age (NS; p<0.27).

### Association between the levels of IGF1 and fracture toughness is influenced by age

**Fig 4:**
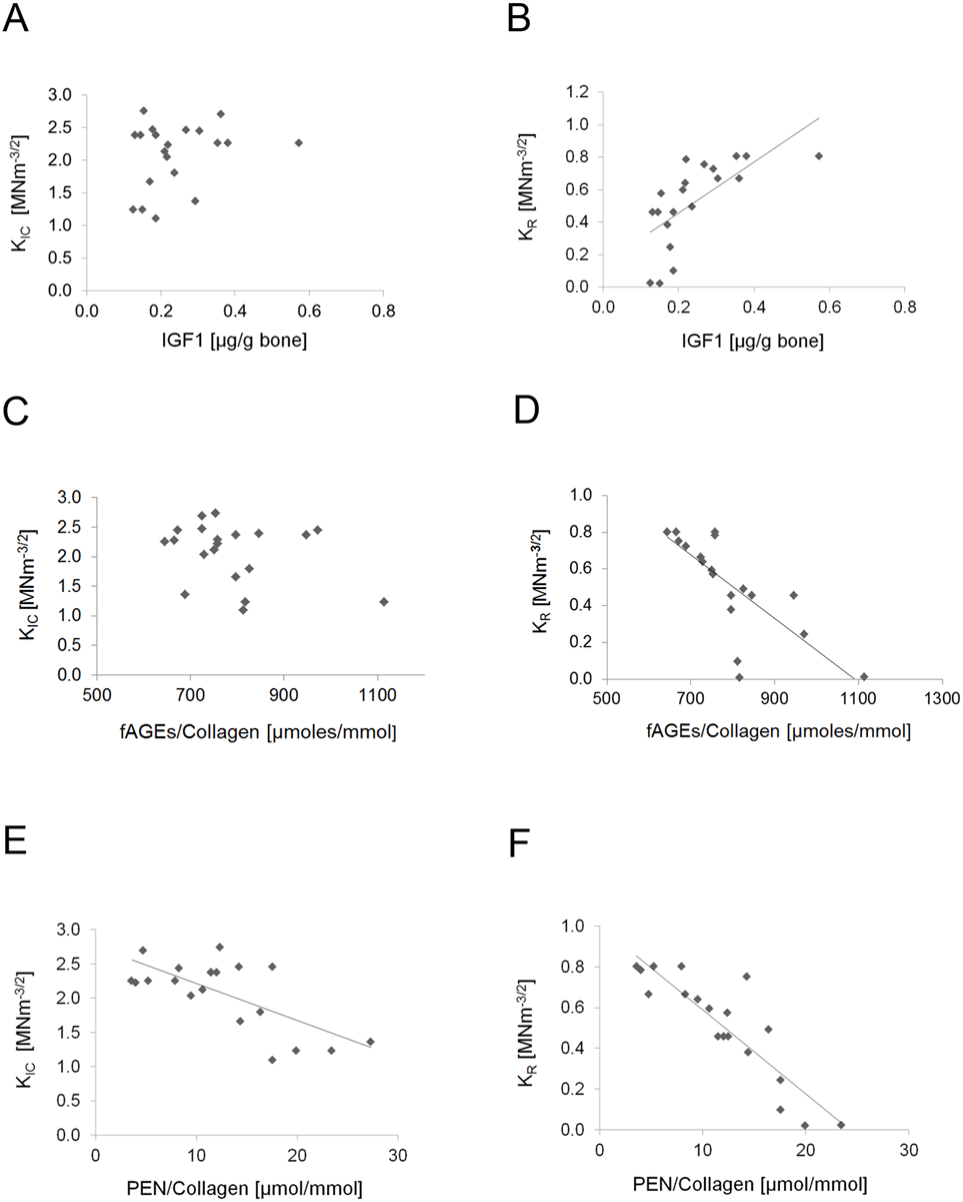
Initiation (K_IC_) and propagation (K_R_) toughness of human bone in relation to the levels of IGF1, collagen and pentosidine. **A**. There is no significant correlation between the IGF1 levels and initiation toughness. **B**. Bones with high concentration of IGF1 show higher propagation toughness (simple linear regression only p<0.001). **C**. There is no significant correlation between the fAGEs levels and initiation toughness. **D**. Propagation toughness shows significant relationship with the levels of fAGEs per mmol of collagen (simple regression: p<0.001; multiple regression: p<0.01). PEN concentration in bone shows some relationship with propagation toughness (simple linear regression p<0.001 only). **E**. PEN concentration in bone displays a significant relationship with initiation toughness (simple linear regression p<0.001; multiple linear regression p<0.01). **F**. PEN concentration in bone shows significant relationship with propagation toughness (simple linear regression p<0.001) that is lost after adjustment for age (p>0.05).

We determined that there was no significant correlation between initiation toughness and concentration of IGF1 in bone (Fig. 4*A*). Positive and highly correlated relationship between IGF1 concentration and propagation toughness of bone was observed for simple linear regression (Fig. 4*B*; simple linear regression R^2^ = 0.45, p<0.001 with IGF1 as independent variable and propagation toughness as dependent variable), but not by multiple linear regression with IGF1 and age as independent variables and propagation toughness as dependent variable). Bones with high concentration of IGF1 in bone (0.713 μg/g bone; average calculated from the values of three independent measurements) showed higher propagation toughness (average 0.79 MNm^-3/2^) as compared to those with lower concentration of IGF1 in bone (average 0.223 μg/g bone and average 0.38 MNm^-3/2^, respectively; average calculated from the values of three independent measurements).

### Relationships between IGF1, glycation and fracture toughness

Using simple linear regression analysis, we established that although there was no association between fAGEs and initiation toughness (Fig. 4*C*), there was a significant association between propagation toughness and fAGEs (Fig. 4*D*, R^2^ = 0.50, p<0.001). Simple linear regression analysis revealed the potential influence of PEN modification on the initiation (Fig. 4*E*; R^2^ = 0.45, p<0.01) and propagation (Fig. 4*F*; R^2^ = 0.79, p<0.001) toughness of human cortical bone. The multiple linear regression models using IGF1, fAGEs, PEN and age as independent variables to predict initiation or propagation toughness indicated that initiation toughness was influenced only by PEN (p = 0.01; R^2^ = 0.56), whereas propagation toughness was influenced by fAGEs and age (p < 0.05; R^2^ = 0.90) (Table 1).

**Table 1:** Summary of the results from the multiple linear regression analysis.

Parameter	K_IC_	K_R_
Intercept	NS	0.0161
IGF1	NS	NS
fAGEs	NS	0.0150
PEN	0.0129	NS
Age	NS	0.0010
R^2^	0.56	0.90

## Discussion

Of particular interest for this study is IGF1 and its potential role in glycation and bone fragility. We hypothesized that IGF1 may function as one of the metabolic factors that contribute to glycation/AGEs-formation and fragility fractures, and this has not been proposed before.

The selected cortical bone from posterior cortex region of tibia was of our specific interest, because in addition to the IGF1-dependent regulation of cortical bone size [1, 3], cortex makes up 80% of the skeleton and cortical bone is preferentially compromised in diabetic patients (type 2 diabetes) [41]. Fractures are newly recognized complication of diabetes. Our work on healthy, normally aging donors provides the foundation, for example, for studies on the role of IGF1, glycation and bone fragility in aging and diabetic human populations and animal models.

To perform fracture toughness testing, we used compact tension specimens prepared from human tibias. Compact tension specimens are particularly useful for studying fracture mechanics of bone, because not only their geometry facilitates controlled crack propagation for a range of applied strain rates, but also closely follows principles of fracture mechanics. In addition, the geometries of the compact tension specimen and of the notch influence the rate of total energy dissipation. Through this energy dissipation the connection between fracture mechanics and extracellular matrix can be determined [8].

To analyze fracture toughness of bone in relation to several biochemical parameters of bone matrix, we determined concentrations of IGF1, fAGEs and PEN. The measured levels and age-related decreases of IGF1 in human cortical bone of tibia were similar to the ones observed by other investigators for human iliac crest. Thus, the highest and the lowest amounts of IGF1 observed in human iliac crest samples were 0.76–0.87 μg/g bone and 0.08–0.24 μg/g bone, respectively, and the IGF1 decline was approx. 1.5-fold between third and eight decade of human life [30]. Due to the analysis of a larger age range of the donors (two decades more) of a different skeletal site, we observed a more pronounced decline of IGF1 with age than previously reported [30].

The accumulation of fAGEs and PEN per collagen increased with the donors’ age (Fig. 3*B* and *D*) and displayed inverse relationship with the IGF1 concentration (Fig. 3*C* and *E*). To determine to what degree the observed associations between IGF1, fAGEs and PEN levels in bone matrix were influenced by age, we performed the multiple linear regression analysis. This analysis confirmed the significant association of IGF1 with fAGEs. Interestingly, the association between IGF1 and PEN was slightly influenced by age. PEN is considered to be well-defined AGE of sugar origin. In the first step of PEN formation, the attachment of the aldehyde group of open-chain glucose to free amino groups of amino acids (lysines or arginines) depends on the overall glucose levels, which are influenced by different factors including IGF1 [31–33]. In the last step of PEN formation, the conversion of pentosidine precursor(s) into mature PEN involves oxidation [22, 42, 44]. Since oxidation processes increase the conversion of pentosidine precursor, pentosinane, into mature PEN [22], they can skew the association between IGF1 and PEN. In summary, the increase of fAGEs coinciding with the decline of IGF1 in bone indicates the potential association between IGF1 and glycation.

One of the possible explanations for the observed association between IGF1 and glycation could be the involvement of the insulin/IGF1 signaling pathway [43]. Reducing the level of the insulin/IGF1 signaling extends lifespan, delays the onset of age-related diseases and reduces their severity, suggesting that this signaling pathway couples the normal aging process to age-related disease susceptibility [43–45]. Population studies confirm that average blood glucose levels in the fasting state increase with age [46–48]. This blood glucose gradient is statistically significant even when confounding factors, such as obesity are considered. Steady increase of the fasting glucose level over a lifetime is a different issue than the increase of glucose resistance and the decline in insulin secretion due to aging [46]. Interestingly, blood glucose levels in humans associate with life-span and they increase much slower for long-lived individuals [46, 48]. This suggests that there may be involvement of the insulin/IGF1 signaling pathway in the association of IGF1 and glycation.

After accounting for age, we observed that PEN correlated to initial toughness and no significant relationship between initiation toughness and concentration of IGF1 in bone (Fig. 4*A*). In contrast, positive significant relationship was noted for fAGEs with propagation but not initiation toughness. This difference in correlation between the two measures (fAGEs, PEN) and initiation vs. propagation toughness most likely represents a greater accumulation of AGEs in bone other than PEN that affect the overall fracture of bone. We have previously showed that, in contrast to PEN, fAGEs predict the post-yield energy to fracture [29].

Given the complexity of the IGF1 functions, the aforementioned results can be best explained in the context of the stimulatory effect of IGF1 on glucose metabolism, collagen synthesis and the corresponding increase in the bone matrix production [49]. With aging and diabetes, increased glucose levels would enhance bone matrix glycation, while impairing both collagen turnover and matrix renewal. As a result, normal bone formation would be impaired and this would lead to fragile bones as demonstrated by the lower toughness values in bones containing higher level of AGEs per gram of collagen (Fig. 4*F*). Collagen modifications stiffen bone matrix and modify the fracture behavior of bone leading to an instantaneous and brittle bone fracture under physiological levels of strain [8, 21].

In addition to collagen, there are approx. six to eight major bone matrix proteins [38, 50]. Osteocalcin and osteopontin have recently begun to be recognized as critical determinants of bone quality due to their ability to resist fracture [7, 38]. Taken together, collagen and non-collagenous proteins (NCPs) are important contributors to bone quality [7] as both these groups of proteins are subject to different non-enzymatic (e.g., undesired glycation) and enzymatic post-translational modifications. Enzymatic trivalent mature crosslinks such as pyridinoline (PYD) and deoxypyridinoline (DPD) are the predominant stabilizing inter- and intra-molecular crosslinks in collagen. Although relatively ubiquitous, PYD dominates in cartilage, whereas DPD is more specific for bone [12]. In adult bone, the PYD and DPD concentrations are lower in trabecular than cortical bone [10, 18]. We did not observe any abnormal levels of PYD and DPD in the analyzed bone samples that originated from healthy, normally aging human donors (S2*A* and *B* Fig.).

**Fig 5:**
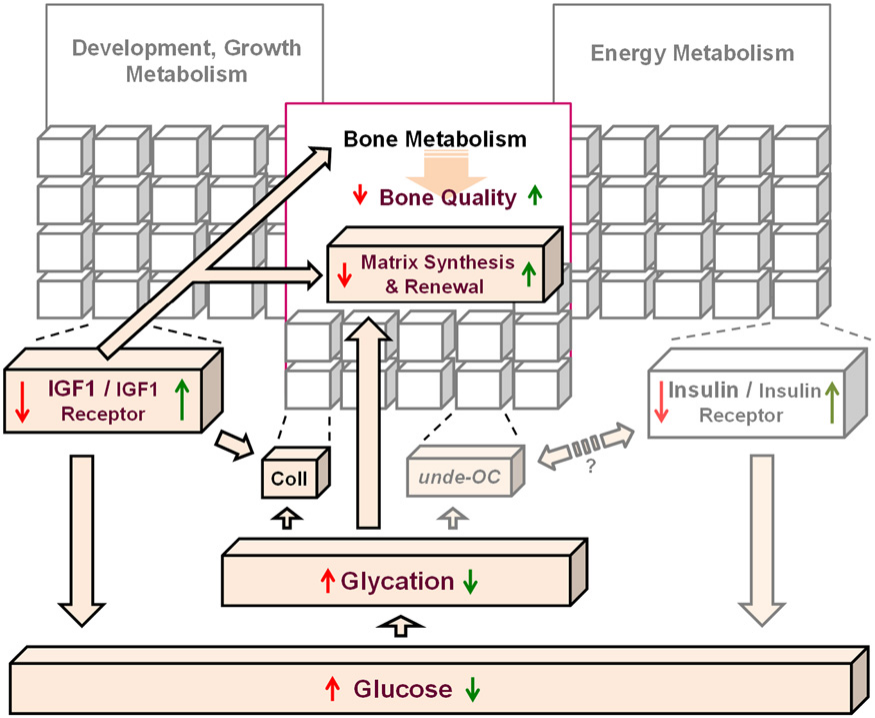
Schematic showing the investigated associations between IGF1 levels, glycation products and bone quality (indicated by the dark colors). The effects of positive (green arrows) or negative (red arrows) changes of the IGF1 levels in bone tissue are shown by arrows.

Due to the fundamental importance of maintaining a healthy glucose/energy metabolism, an array of hormones and morphogenetic proteins evolved in vertebrates to control and tune the glucose/energy metabolism (shown as gray cubes in Fig. 5). Therefore, it is a major challenge to determine the nature of the interactions within the investigated IGF1/sugar metabolism/bone quality axis. The complexity of IGF1 functions and the role of the lifestyle and environmental factors in the aforementioned axis are still poorly understood. During evolution, a single insulin/IGF1 pathway diverged into two hormonal pathways in mammals; insulin evolved to perform primarily a metabolic role in energy metabolism, while IGF1/growth hormone axis evolved to serve growth, development, and likely, longevity [33]. Therefore, the contemporary model assigns IGFs a central role in regulating growth, development and reproduction in mammals, while insulin serves to regulate energy accumulation, storage and expenditure. However, IGF1 and insulin exert overlapping roles in many physiological processes [31, 32]. Animal studies using a variety of species, including nondiabetic and diabetic (spontaneous and pancreatectomized) animals, have demonstrated that IGF1 lowers glucose levels in blood (i.e., hypoglycemic activity of IGF1) by stimulating glucose uptake from the bloodstream [49–51].

We propose that the increased/high levels of IGF1 during development as well as growth and puberty (Fig. 5, green arrows) exert desired effects on bone quality (considered as the sum of total characteristics of bone), and thus, protect bone from fracture. Conversely, decreased/low levels of IGF1 lead to poor (red arrows) bone quality. Thus, due to its involvement in glucose metabolism [30, 31, 49–51, 53–55] and the reciprocal cross-talk between the IGF1- and insulin- receptors, IGF1 may play an important role in the age-related accumulation of AGEs in bone tissue (Fig. 5).

Interestingly, osteocalcin was recently identified as an osteoblast-secreted hormone regulating insulin secretion and sensitivity [32, 52]. Osteocalcin (OC) is a part of a complex signaling network between bone and the organs classically associated with the regulation of energy homeostasis, such as the pancreas and adipose tissue. OC’s function is regulated by insulin and leptin [56]. In mice and humans, osteocalcin can be present in the serum in both carboxylated and undercarboxylated (unde-OC) form. It has been shown that in mice the undercarboxylated form of osteocalcin acts as a hormone integrating bone with energy metabolism and alters insulin expression, secretion and sensitivity [32, 52, 56]. Delineation of the role of carboxylated and undercarboxylated osteocalcin in glucose metabolism in humans is difficult, because it is obscured by many factors (e.g., age, diet, circadian rhythm, medications, physical activity, etc.), in particular, by vitamin-K-dependent gamma-carboxylation of glutamic acid residues [57]. Systematic investigations are required to get insight into these important processes.

Since bone matrix quality depends on healthy matrix renewal and synthesis, and is negatively influenced by glycation, the age-related deterioration of the IGF1/sugar metabolism/bone quality axis may have a negative impact on bone due to the increased matrix glycation and impaired collagen turnover. In addition, the association between IGF1 and AGEs levels could also be influenced by the accumulation of collagen-bonded glycation products when bone turnover slows down. Further studies are needed to elucidate these complex metabolic processes.

## Conclusions

To our knowledge, this is the first study showing the relationship between matrix level IGF1 and bone fracture as well as the association of IGF1 levels with the levels of glycation products. The link between IGF1-related formation of AGEs and resistance of bone to fracture may have important implications for understanding the role of protein matrix in bone toughness as well as in the development of new diagnostic tools and treatments for osteoporosis or diabetes. Our studies lay the groundwork for broad investigations of the association between IGF1, AGEs and bone quality in vertebrates.

## Supporting Information

S1 Fig Examples of the ultra-performance liquid chromatography (UPLC) chromatograms. Detection of pentosidine (PEN) in the analyzed bone samples as compared to the PEN standard. **B**. Detection of *L*-hydroxyproline (ProOH) in the analyzed bone samples as compared to the ProOH standard. Collagen amount in a given sample was calculated as described by Sroga and Vashishth [2011].
(DOCX)

S2 Fig Concentration levels of pyridinoline (PYD) and deoxypyridinoline (DPD) in the analyzed bone samples that originated from healthy, normally aging human donors. **A**. Age-related levels of PYD/collagen. **B**. DPD levels per collagen. Our data confirm that the content of PYD and DPD reaches a maximum concentration between 15 and 30 years of age, and then, is maintained at an approx. constant level until the age of late 80’s. In general, human cortical bone displayed approx. 3–4-fold higher level of DPD over PYD.
(DOCX)
