# Supplementary material for: Insulin-Like Growth Factor 1, Glycation and Bone Fragility: Implications for Fracture Resistance of Bone
Source: PLoS One. 2015 Jan 28;10(1):e0117046. doi: 10.1371/journal.pone.0117046 (PMC4309541; doi:10.1371/journal.pone.0117046)
Supplement: S1 Fig — Detection of pentosidine (PEN) in the analyzed bone samples as compared to the PEN standard. B. Detection of L-hydroxyproline (ProOH) in the analyzed bone samples as compared to the ProOH standard. Collagen amount in a given sample was calculated as described by Sroga and Vashishth [2011]. (DOCX) [file pone.0117046.s001.docx]

**Figure S1**

A

B
