# Supplementary figures and images for: Insulin-Like Growth Factor 1, Glycation and Bone Fragility: Implications for Fracture Resistance of Bone
Source: PLoS One. 2015 Jan 28;10(1):e0117046. doi: 10.1371/journal.pone.0117046 (PMC4309541; doi:10.1371/journal.pone.0117046)

**Figure S2**

B

A


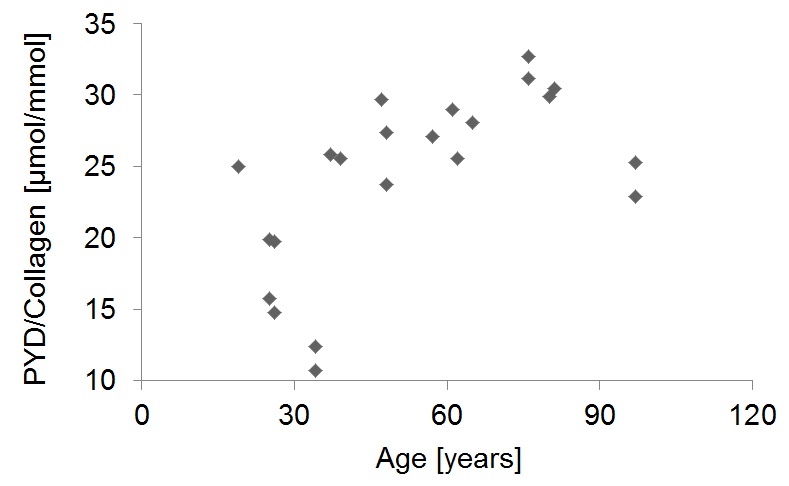

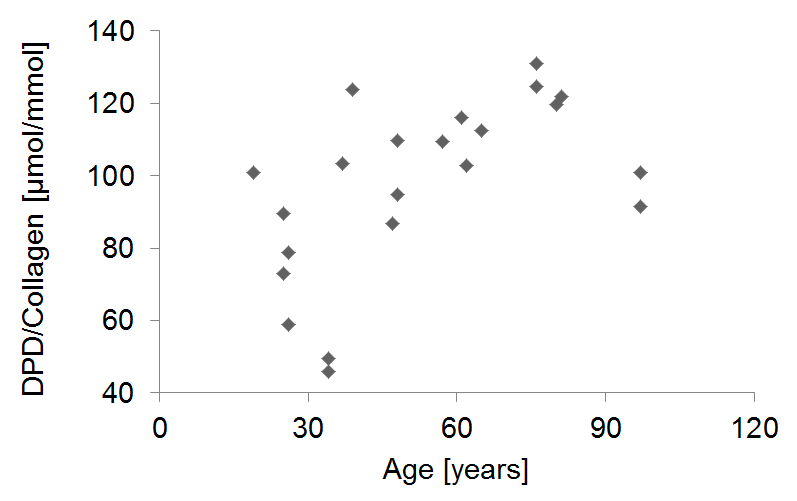

Supplement: S2 Fig — A. Age-related levels of PYD/collagen. B. DPD levels per collagen. Our data confirm that the content of PYD and DPD reaches a maximum concentration between 15 and 30 years of age, and then, is maintained at an approx. constant level until the age of late 80’s. In general, human cortical bone displayed approx. 3–4-fold higher level of DPD over PYD. (DOCX) [file pone.0117046.s002.docx]
